# HEALTH DISPARITIES RELATED TO COST OF MEDICAL COVERAGE

**DOI:** 10.1093/geroni/igac059.2846

**Published:** 2022-12-20

**Authors:** Nathaniel Palmer, Laura Bernstein, Julie Hicks Patrick

**Affiliations:** West Virginia University, Morgantown, West Virginia, United States; West Virginia University, Morgantown, West Virginia, United States; West Virginia University, Morgantown, West Virginia, United States

## Abstract

Older age is often associated with challenges to functional ability. There are many government and private programs in place to help support the functioning of adults, including Medicaid, Medicare, and Tricare. These programs are not always effective, especially for middle aged and older adults. We examined whether cost was a barrier to receiving care and whether health insurance status acted as moderators of the relationship between age and functional ability. We used data from 379,606 adults who completed the 2020 Behavior Risk Factor Surveillance System (median age = 54.4 years; 54.2% women; 75.3% white non hispanic). The moderated regression equation was significant, F (7, 379598) 3275.74, p < .001, R^2 = .057. Although direct effects of age emerged as significant, the relation on functional ability was qualified by two 2-way interactions: age by insurance (b = .003) and age by prohibitive cost (b = .008). No other effects emerged as significant. Examination of the means revealed that lack of insurance was especially challenging to the functional ability of middle aged adults, whereas cost prohibitiveness was especially challenging for the functioning of older adults. Our results highlight the critical need to research the efficacy of health insurance policies amongst the middle aged and older populations in the United States

